# Increased Resting State Triple Network Functional Connectivity in Undergraduate Problematic Cannabis Users: A Preliminary EEG Coherence Study

**DOI:** 10.3390/brainsci10030136

**Published:** 2020-02-28

**Authors:** Claudio Imperatori, Chiara Massullo, Giuseppe Alessio Carbone, Angelo Panno, Marta Giacchini, Cristina Capriotti, Elisa Lucarini, Benedetta Ramella Zampa, Eric Murillo-Rodríguez, Sérgio Machado, Benedetto Farina

**Affiliations:** 1 Cognitive and Clinical Psychology Laboratory, Department of Human Science, European University of Rome, Italy, Via degli Aldobrandeschi 190, 00163 Roma, Italy; chiara_massullo@yahoo.it (C.M.); giuseppea.carbone@gmail.com (G.A.C.); angelo.panno@unier.it (A.P.); marta.giacchini@hotmail.com (M.G.); cristinacap1996@hotmail.it (C.C.); elisalucarini1941@gmail.com (E.L.); b.ramellazampa@gmail.com (B.R.Z.); benedetto.farina@unier.it (B.F.); 2 Laboratorio de Neurociencias Moleculares e Integrativas, Escuela de Medicina, División Ciencias de la Salud, Universidad Anáhuac Mayab, Mérida 97302, Yucatán, Mexico; eric.murillo@anahuac.mx; 3 Laboratory of Physical Activity Neuroscience, Physical Activity Sciences Postgraduate Program–Salgado de Oliveira University (UNIVERSO), Niterói 24030-060, RJ, Brazil; secm80@gmail.com

**Keywords:** problematic cannabis use, triple network, EEG functional connectivity, eLORETA, resting state

## Abstract

An increasing body of experimental data have suggested that aberrant functional interactions between large-scale networks may be the most plausible explanation of psychopathology across multiple mental disorders, including substance-related and addictive disorders. In the current research, we have investigated the association between problematic cannabis use (PCU) and triple-network electroencephalographic (EEG) functional connectivity. Twelve participants with PCU and 24 non-PCU participants were included in the study. EEG recordings were performed during resting state (RS). The exact Low-Resolution Electromagnetic Tomography software (eLORETA) was used for all EEG analyses. Compared to non-PCU, PCU participants showed an increased delta connectivity between the salience network (SN) and central executive network (CEN), specifically, between the dorsal anterior cingulate cortex and right posterior parietal cortex. The strength of delta connectivity between the SN and CEN was positively and significantly correlated with higher problematic patterns of cannabis use after controlling for age, sex, educational level, tobacco use, problematic alcohol use, and general psychopathology (*r*_p_ = 0.40, *p* = 0.030). Taken together, our results show that individuals with PCU could be characterized by a specific dysfunctional interaction between the SN and CEN during RS, which might reflect the neurophysiological underpinnings of attentional and emotional processes of cannabis-related thoughts, memories, and craving.

## 1. Introduction

Cannabis is the most widely used illicit drug in Europe, with 18% and 9.3% of young people (i.e., the 15–24 age group) reporting having used cannabis in the last year and in the last month, respectively [1]. The lifetime prevalence of cannabis use disorder is about 6% [2], and the frequency of patients being treated for the first time for cannabis problems has dramatically increased over the last decade [1]. Therefore, cannabis use is considered a relevant topic that is gaining greater attention not only from a political point of view [1] but also from a scientific point of view, with a specific focus on the cognitive, behavioral, and neurobiological consequences associated to its use and abuse [3].

For example, research on animal models documented that while high concentrations of Δ9-tetrahydrocannabinol (THC), the main psychoactive constituent of cannabis [4], are necessary to impair memory and cognition in old rats, even low concentrations are deleterious in young animals [5]. Animal studies also showed that chronic THC exposure is associated with widespread neurochemical and neuroanatomical alterations in several brain areas, such as the limbic system and prefrontal cortex [6].

Similarly, human neuroimaging studies have shown that problematic cannabis use is related to different structural, functional, and neurophysiological brain alterations [7]. For instance, structural neuroimaging studies showed abnormalities in hippocampus volume and gray matter density associated with cannabis use [8]. Furthermore, Moreno-Alcázar et al. [9] recently reported that, compared to healthy controls, long-term heavy cannabis users showed increased gray matter volume in the basal ganglia and nucleus accumbens. A recent meta-analysis [3] on 35 task-related functional imaging studies also showed that cannabis use is associated with a decreased activity in brain areas involved in cognitive control process (e.g., the anterior cingulate cortex and dorsolateral prefrontal cortex (dlPFC)) and increased activity in brain structures involved in reward processing (e.g., the striatum). Lastly, electroencephalographic (EEG) studies showed that cannabis use is related to several neurophysiological abnormalities, such as increased cortical activation and connectivity, not only during drug cue exposure [10,11,12] but also during resting state (RS) condition [13,14,15].

Taken together, all these data are in line with the perspective that reward-related behaviors and addictive disorders are associated with dysfunctional dynamic interactions between large neural networks rather than alterations in single brain areas [16,17,18,19]. Within this modern view of the brain as a highly integrated and dynamic system, in the last years, a theoretical model has gained particular attention in the neuroscientific literature, the so-called triple network model [20]. This conceptualization underlines the crucial role of the synergistic interaction between large-scale networks in regulating the general access to cognitive functions [21] and conversely, it suggests that the dysfunctional communication within these neural systems is the most plausible explanation of psychopathology across multiple mental disorders [20,22]. 

In particular, the triple network model [20] focuses on the dynamic interaction among the default mode network (DMN), salience network (SN), and central executive network (CEN). While the DMN, centered on nodes in the medial prefrontal cortex (mPFC) and posterior cingulate cortex (PCC), is typically active during RS and involved in several higher-order integrative mental functions such as self-referential processing and mentalization [23,24], the CEN, anchored bilaterally in the dlPFC and posterior parietal cortex (PPC), is typically active during a wide range of cognitive tasks and involved in several mental functions such as working memory and problem solving [20,21]. The functional and dynamic switch between the DMN and CEN (i.e., between task-based and task-free states) is assured by the regular activity of the SN [25,26], which includes the dorsal anterior cingulate cortex (dACC) and bilateral anterior insula [20]. Indeed, this network plays a crucial role in filtering, detecting, and integrating relevant internal (e.g., autonomic input) and external (e.g., emotional information) salient stimuli in order to guide behavior [27,28].

In the last decade, an increasing body of experimental data has suggested that different aberrant functional interactions among the SN, CEN, and DMN may be considered potential neurophysiological biomarkers of different psychopathological phenomena emerging across several neuropsychiatric disorders, including substance-related and addictive disorders [20,22,29].

For example, it has been reported that, compared to the smoking state, nicotine abstinence is associated with lower SN–DMN connectivity, suggesting that a weaker network interaction contributes to smoke craving [30]. Decreased connectivity between the SN and DMN was also reported in cocaine-dependent individuals [31,32]. Furthermore, Li et al. showed that greater connectivity between the SN and DMN, as well as lower connectivity between the CEN and DMN, is associated with relapse behavior in heroin-dependent patients [33].

To the best of our knowledge, only one report has explored the association between cannabis use and triple network connectivity. In a functional magnetic resonance imaging (fMRI) study, Wall et al. [34] showed that in recreational cannabis users (i.e., not regular users) THC administration disrupts the DMN, where the PCC was the key brain region involved in the subjective experience of THC intoxication. Thus, the primary purpose of the current research was to extend these previous results examining the association between problematic cannabis use (PCU) and triple network EEG functional connectivity. Indeed, although fMRI is widely used to investigate brain functional connectivity, EEG is considered a suitable tool to investigate network properties [35,36], providing relevant data on functional interactions between dynamic neural systems in each frequency band [37,38].

## 2. Materials and Methods

### 2.1. Participants

Study participants were enrolled using advertising material posted around the university campus (i.e., a brief explanation of the study procedure including EEG procedure and questionnaire administration). The enrolment lasted from September to December 2019. Ninety-five undergraduate students who agreed to participate were screened for eligibility. All the individuals provided informed consent and contributed voluntarily to the study (i.e., they did not receive payment or academic credits). This research was approved by the ethics committee of the European University of Rome (Prot. N.008/19) in line with the Helsinki declaration standards.

Twelve participants (7 males and 5 females) with problematic cannabis use (PCU group) and twenty-four (9 males and 15 females) non-cannabis-using participants (non-PCU group) were finally enrolled. PCU individuals were enrolled if they met the following inclusion criteria: (i) Cannabis Abuse Screening Test (CAST) [39] total score ≥7, as recommended by Bastiani et al. [40] (see “self-report measures” section for details); (ii) frequency use of cannabis during the last 12 months ≥20 times [40]; (iii) age range 18–30 years old; (iv) negative past or current diagnosis of any psychiatric and/or neurological diseases (including head trauma); (v) right-handedness; (vi) negative psychoactive medications use and other illegal drugs consumption in the past two weeks prior to the EEG recordings. 

Non-PCU group were included if they met the following inclusion criteria: (i) CAST total score = 0; (ii) frequency use of cannabis during the last 12 months = 0 times; (iii) age range 18–30 years old; (iv) negative past or current diagnosis of any psychiatric and/or neurological diseases (including head trauma); (v) right-handedness; (vi) negative psychoactive medications use and other illegal drugs consumption in the past two weeks prior to the EEG recordings.

### 2.2. Self-Report Measures

After the enrolment, all subjects were administered the CAST [39], a self-report measure of alcohol use problems (CAGE) [41], and the Symptom-Checklist-K-9 (SCL–K–9) [42], and they were asked screening questions according to a checklist developed for previous studies [43,44,45,46].

The CAST [39] is a 6-item self-report questionnaire widely used to assess problematic patterns of cannabis use within the past 12 months [47]. Items are scored on a 5-point Likert scale (from 0 = “never” to 4 = “very often”). The CAST includes two scoring options [48,49]: a binary version (i.e., computing the positive response thresholds that vary across items) and a full version (i.e., calculating the score using the full range of item responses). Good psychometric properties (e.g., high internal consistency) of both versions have been reported [48,49]. Satisfactory cross-cultural adaptation has been also documented [50,51]. In a sample of Italian young adults, using the Multiple Correspondence Analysis (MCA), Bastiani et al. [40] maximized item homogeneity of the CAST and obtained the best score in relation to the importance of the response categories for each item. Using this procedure, the authors showed that, compared to both the binary and the full version, the CAST MCA form had better psychometric properties and that the optimal cut-off score was 7 [40]. Therefore, in the current study, the CAST MCA version was used, and the Cronbach’s α in our sample was 0.91.

The CAGE [41] is a 4-item self-report widely used to assess problematic alcohol use [41,52]. The acronym refers to the 4 dichotomous (yes = 1; no = 0) questions investigated by the questionnaire: (i) Cut down, (ii) Annoyed, (iii) Guilty, and (iv) Eye. The total score ranges from 0 to 4, and the recommended cut-off to screen problematic alcohol use is ≥2 [53]. Previous researches [53] reported that the CAGE has satisfactory psychometric properties (e.g., suitable correlations with other screening instruments). In the current research, we used the Italian adaptation of the CAGE [54], and the Cronbach’s α in our sample was 0.68.

The Symptom-Checklist-K-9 (SCL–K–9) [42] is the short unidimensional version of the original Symptom Checklist-90-Revised (SCL–90–R) [55]. It is composed of the nine items of the SCL-90-R (rated on a 5-point Likert scale ranging from 0 = “not at all” to 4 = “extremely”), showing the highest discriminant power with the general level of psychopathology (i.e., the global severity index). Good psychometric properties (e.g., good reliability and good model fit), as well as significant correlations with other questionnaires assessing psychological distress, have been reported [56]. In the present study, we used the Italian adaptation of the SCL-K-9 [57], and the Cronbach’s α in our sample was 0.86.

### 2.3. EEG Data Acquisition and Functional Connectivity Analysis

All EEG recordings were performed in the Cognitive and Clinical Psychology Laboratory of the European University of Rome. Eyes-closed RS EEG was recorded for at least 5 minutes. Study participants were invited to sit comfortably with their eyes closed in a quiet, semidarkened silent room; subjects were also instructed to avoid alcohol, caffeine, and cigarettes immediately before their experimental session (i.e., at least 4 h).

EEG data acquisition was performed using Micromed System Plus digital EEGraph (Micromed© S.p.A., Mogliano Veneto, TV, Italy) and 31 standard scalp leads, placed according to the 10-20 system. In this setting, Electro-oculogram and the Electrocardiogram were also acquired, and the reference electrodes were placed on the linked mastoids. As regards the EEG signal, it has been used a sampling frequency of 256 Hz and impedances were kept below 5KΩ before starting the recording and further controlled at the end of each experimental session. Other details about EEG recordings (e.g., A/D conversion and preamplifiers amplitude) can be found elsewhere [58,59]. Signal processing (i.e., filtering and artifact rejection procedure) was performed using EEGlab toolbox for MATLAB (The MathWorks, Inc). For filtering procedure, the “basic FIR filter” option was selected, and 0.2 Hz and 100 Hz were respectively the high-frequency filter and the low-frequency filter. Artifact rejection (i.e., removal of eye movements, blinks, cardiac pulses, muscular or movement activities) was performed visually on the raw EEG (for details, see [59,60,61]). At least 3 minutes of clean EEG data (not necessarily consecutive) were selected and analyzed for each subject. According to previous exact Low-Resolution Electromagnetic Tomography software (eLORETA) studies [43,45,62,63,64,65,66], artifact-free data were fragmented into epochs of 2 seconds for the EEG coherence analysis.

The exact Low-Resolution Electromagnetic Tomography software (eLORETA), a well-corroborated computer program able to detect electrocortical activity [67], was used for all EEG analyses. The eLORETA provides a “discrete, three-dimensional (3D) distributed, linear, weighted minimum norm inverse solution” [62]. Assuming that adjacent neuronal sources will be highly synchronized, the exact weights used in this software “endow the tomography with the property of exact localization to test point sources, yielding images of current density with exact localization albeit with low spatial resolution” [62]. The head model for the inverse solution uses the electric potential lead field computed with the boundary element method [68] averaged of a magnetic resonance image (MRI) data set. This forward equation “corresponds to an instantaneous discrete sampling of the measurement space (scalp electrodes) and the solution space (cortical voxels)” [67]. In other words, computations were performed using a realistic head model [68] determined according to the digitized MNI152 template provided by the Brain Imaging Center of the Montreal Neurological Institute (MNI) [69]. The standard electrode locations on the MNI152 scalp have been determined according to previous studies [70,71]. The three-dimensional spatial solution is limited to cortical gray matter, as determined by the probabilistic Talairach atlas [72], comprising 6239 voxels of 5 cubic mm spatial resolution (for details, see [62,63,64,73,74]). Only voxels that were unambiguously identified as cortical grey matter and those unequivocally felled within the brain compartment were considered by the software. Therefore, eLORETA images reflect the exact electrocortical activity at each voxel in neuroanatomic MNI space as the exact magnitude of the estimated current density [75]. Although the computations should be ideally performed on the exact head model, determined from each individual subject’s MRI, the boundary element method is considered a suitable technique and it is one of the often-used realistic models in EEG source analysis [76]. Furthermore, compared to the previous version (e.g., sLORETA), the eLORETA is characterized by a correct localization even in the presence of structured noise [67,74]. Previous reports showed that the eLORETA provides a suitable localization agreement (the average depth localization error was 7 mm) with other neuroimaging methods [77,78,79,80,81,82,83], and also when a low number of electrodes were used (i.e., <30). The eLORETA is also characterized by no localization bias even in the presence of structured noise [62,67,84]. This software is also considered a suitable tool to investigate large brain network dynamics [35,36,38] by evaluating the modifications in the neuronal synchronization at varying time delays and frequencies [36]. As a matter of fact, compared to other brain-imaging methods, EEG time-series data provide a direct measure of postsynaptic potentials with millisecond temporal resolution [38,84], providing a relevant and precious complementary source of data for scholars and practitioners in a relatively ecological and economical way [85,86].

In the present study, the lagged phase synchronization (LPS) method [67,87] was used in order to investigate functional connectivity. The LPS evaluates “the similarity of two time series by means of the phases of the analyzed signal” [88] based on normalized Fourier transforms [63] with values ranging from 0 (i.e., no synchronization) to 1 (i.e., the maximum synchronization). Therefore, this approach is related to nonlinear functional connectivity, and it is considered to be accurately corrected, representing the synchrony of two signals after the removal of the instantaneous zero-lag component, which is characterized by several artifacts, such as volume conduction [63]. Although removing zero-lag phase synchronization could not completely remove volume conduction [89], the LPS is considered to include only physiological connectivity information and, compared to other connectivity indexes, it is also minimally affected by low spatial resolution [63,67]. For these reasons, the LPS is broadly used in clinical neurophysiology studies [62,63,64,65,88,90,91,92]. 

According to Li and coworkers [33], the triple network functional connectivity was investigated defining

**Figure 1:**
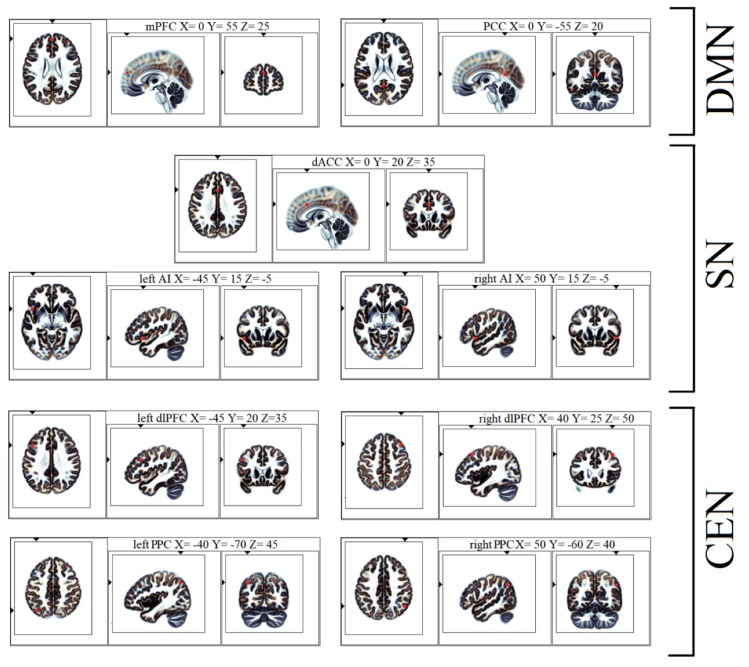
eLORETA ROIs of the triple network and Montreal Neurological Institute coordinates (Axial, Sagittal, and Coronal view). Abbreviations: eLORETA = exact Low Resolution Electromagnetic Tomography software; ROIs = Regions of Interests; mPFC = medial prefrontal cortex; PCC = posterior cingulate cortex; DMN = default mode network; dACC = dorsal anterior cingulate cortex; AI = anterior insula; SN = salience network; dlPFC = dorsolateral prefrontal cortex; PPC = posterior parietal cortex; CEN = central executive network.

9 Regions of Interest (ROIs; Table 1 and Figure 1). The LPS was calculated between all the ROIs (i.e., 81 connections) by the eLORETA, which also performed the source reconstruction [93,94]. According to previous reports [67,84], the “single nearest voxel” option (i.e., each ROI consisted of a single voxel, the closest to each seed) was chosen. In the current research, the following frequency bands were analyzed: delta (0.5–4 Hz); theta (4.5–7.5 Hz); alpha (8–13 Hz); beta (13.5–30 Hz); and gamma (30.5–60 Hz).

### 2.4. Statistical Analysis

EEG connectivity analyses were compared between PCU group and non-PCU group, for each frequency band, using the statistical nonparametric mapping (SnPM) methodology available in the eLORETA package. This procedure is based on the Fisher’s permutation [95]. Correction of significance for multiple comparisons (i.e., between all ROIs for each frequency band) was performed using the nonparametric randomization procedure, included in the eLORETA software (for more details, see [64,73]). Briefly, this procedure computes 5000 data randomizations to determine the critical probability threshold of T-values [95,96] corresponding to a statistically corrected (i.e., after the multiple ROIs comparisons in each frequency) *p*-values (*p* < 0.05 and *p* < 0.01). Furthermore, the eLORETA software provides effect size thresholds for t-statistics corresponding to Cohen’s *d* values [97]: small = 0.2, medium = 0.5, large = 0.8. Kolmogorov–Smirnov Z test and chi-squared test were performed to analyze differences between groups for continuous and dichotomous variables, respectively. The association between CAST total score and only statistically significant EEG connectivity data observed in the between-group comparison was evaluated using partial correlation (*r*_p_) analyses, with age, sex, educational level, tobacco use, problematic alcohol use (i.e., CAGE ≥ 2), and SCL-K-9 total score as covariates. IBM SPSS Statistics for Windows, version 18.0 (Chicago, USA), has been used for the statistical analyses.

## 3. Results

For all participants, suitable EEG recordings have been gained. In these recordings, no relevant modifications of the background rhythm frequency (e.g., focal abnormalities or evidence of drowsiness) were detected through a visual assessment of the EEG recordings. The average time analyzed was 248.83 ± 43.58 seconds (Min./Max.: 180/306) and 268.17 ± 38.72 seconds (Min./Max.: 180/318), respectively, for PCU and non-PCU participants (Z-test = 1.02, *p* = 0.252).

Differences between groups are reported in Table 2. No significant differences were observed for socio-demographic data or for general psychopathology, even though, compared to non-PCU, PCU participants reported more frequent tobacco use in the last 6 months, as well as more problematic alcohol use.

### Functional Connectivity Results

In the comparison between PCU and non-PCU participants, the thresholds for significance, corrected for multiple comparisons, were T = ± 3.72 corresponding to *p* < 0.05, and T = ± 4.41, corresponding to *p* < 0.01. The effect sizes for T-threshold were 1.17, 2.92, and 4.67, corresponding, respectively, to small, medium, and large effect sizes.

**Figure 2:**
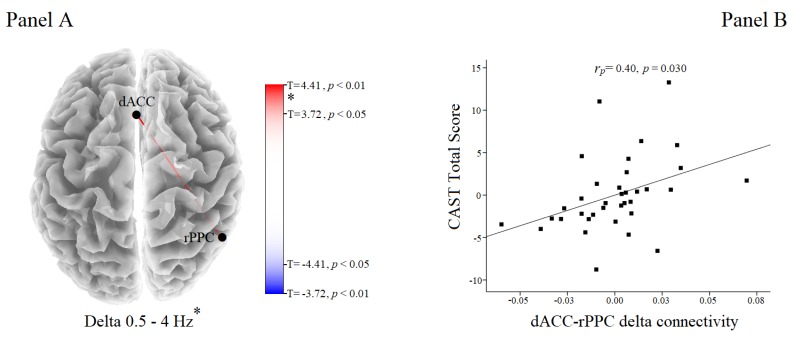
**Panel** (**A**) Results of the eLORETA between comparisons in delta frequency band. PCU individuals showed an increase of delta connectivity (red line) between the dACC and right PPC than non-PCU (T = 4.37, *p* = 0.010). **Panel** (**B**) Scatterplot of the correlation between CAST total score and delta connectivity between dACC and right PPC values adjusted for the effect of potentially competing factors (i.e., sex, age, education level, tobacco use, problematic alcohol use, and SCL-K-9 total score). Abbreviations: dACC = dorsal anterior cingulate cortex; rPPC = right posterior parietal cortex; CAST = Cannabis Abuse Screening Test; SCL-K-9 = Symptom-Checklist-K-9; PCU = problematic cannabis use.

Significant differences between groups were observed in delta band. PCU participants showed an increase of delta connectivity between the dACC and right PPC than non-PCU (T = 4.37, *p* = 0.010; Figure 2A). The strength of delta connectivity between the dACC and right PPC was positively and significantly correlated with the CAST total score after controlling for age, sex, educational level, tobacco use, problematic alcohol use, and general psychopathology (*r_p_* = 0.40, *p* = 0.030; Figure 2B). The correlation between EEG connectivity data and CAST total score remains significant also when the seconds of analyzed EEG were added and considered (*r_p_* = 0.39, *p* = 0.038).

No significant differences were detected in the other frequency bands. The most evident modifications of EEG connectivity observed in the theta band was noticed between the left anterior insula and the PCC (T = 2.45, *p* = 0.61). The most prominent modifications of EEG connectivity observed in the alpha band were reported between the mPFC and the PCC (T = −1.70, *p* = 0.99). The most relevant modifications of EEG connectivity observed in the beta band were detected between the dACC and the right PPC (T = 3.04, *p* = 0.23). Lastly, the most evident modifications of EEG connectivity observed in the gamma band were noticed between the left anterior insula and the PCC (T = 2.67, *p* = 0.45).

## 4. Discussion

The main aim of the current study was to investigate the association between PCU and triple network EEG functional connectivity. Compared to non-PCU, PCU participants showed an increase of delta connectivity between the SN and CEN, specifically, between the dACC and right PPC. Furthermore, SN–CN functional connectivity strength was positively correlated with CAST total score (i.e., higher connectivity was associated with higher problematic patterns of cannabis use), even when controlling for the presence of other variables (i.e., sex, age, educational level, general psychopathology, tobacco use, and problematic alcohol use). No significant association was observed among DMN hubs, suggesting that individuals with PCU could be characterized by a specific dysfunctional communication between the SN and CEN during RS.

In order to support a wide range of cognitive functions, both SN and CEN are conceptualized as task-positive networks interacting with each other [98,99]. Specifically, the SN detects and provides a selective amplification of relevant stimuli generating a top-down control input that activates the CEN in order to respond to salient information [28]. The dACC is considered a key region involved in reward-based decision making, which integrates various task-relevant stimuli and supports goal-directed behavior [100]. Furthermore, it is known that this brain area is crucial during craving-related experiences, not only in response to drug-cues [101] but also during RS condition [78]. On the other hand, the involvement of PPC in a wide range of cognitive tasks, such as attention, decision making, and episodic memory, is well documented [102]. 

Therefore, the increase of RS functional connectivity between the SN and CEN, detected in the present study, might reflect the tendency of PCU individuals to focus on reward-based decision making, triggered by attentional and emotional processes of cannabis-related thoughts, memories, and craving. Accordingly, this study pointed out an increase in SN–CEN connectivity in the delta band. This result is in accordance with previous neurophysiological studies reporting the involvement of delta frequency band in the brain reward system [103,104] and consequently in substance-related disorders, especially during withdrawal and craving states. For instance, the increase of frontal delta and theta power has been reported in crack-cocaine-dependent subjects during guided cocaine imagery [105] as well as in response to acute smoked cocaine self-administration [106]. Similarly, Li et al. [107] reported that delta-increased coherence between frontal and posterior regions was associated with cigarette cravings. Recently, Prashad et al. [13] also showed that cannabis users exhibited a greater cortico-cortical connectivity in both frontal and central regions in delta and theta frequencies band than noncannabis users.

Our results are not consistent with previous studies reporting functional connectivity alterations between DMN hubs and both SN and CEN nodes [30,31,32,33]. These differences could be related to several discrepancies in study designs (e.g., EEG vs. fMRI) and procedures (e.g., ROIs selection). However, it is also possible that specific substances are characterized not only by atypical neurophysiological signatures [13] but also by specific dysfunctional dynamic interactions between neural networks, which might also change according to the different behavioral states (i.e., intoxication, craving, bingeing, withdrawal, and relapse) associated with addiction [108]. This interpretation is purely hypothetical, but it could be investigated in future studies.

Although potentially interesting, the present findings should be evaluated taking into account some limits. The first limitation is the small sample size that reduces the generalizability of our findings and leads us to consider our study only as preliminary. Second, this is a cross-sectional report; thus, causal relationships between investigated variables cannot be established and should be examined through longitudinal and experimental studies. Third, our sample is composed of undergraduate students with no formal diagnosis of cannabis use disorder, which may be characterized by different EEG connectivity patterns within the triple network. Fourth, we did not assess triple network connectivity during drug cues exposure, making our interpretation specific to the RS condition (i.e., eyes closed). Furthermore, although we have excluded participants reporting psychoactive medication use and other illegal drug consumption, a formal urine toxicology screen was not performed. Lastly, it should be noted that abnormalities in grey matter have been reported in PCU, especially in long-term heavy cannabis users [9]. Therefore, although we have investigated young adults with PCU, it cannot completely be excluded that structural alterations might affect the forward modeling by means of different conduction delays and cortical thickness. Notwithstanding these limits, to the best of our knowledge, this is the first study that has examined the association between triple network EEG functional connectivity and PCU using a validated tool (i.e., eLORETA) to localize electrocortical activity and controlling for potential confounding variables.

Based on the results of the current research, future studies should design experimental paradigms using drug, compared to neutral, stimuli to broaden such findings concerning triple network connectivity during direct exposure to drug cues. Moreover, due to the association between PCU and other mental disorders [109], future research considering comorbidity with such disorders is needed to understand relationships among these variables and the neurophysiological mechanisms pointed out through this study. Lastly, future studies with larger samples, longitudinal, and/or experimental designs, and combing multimodal neuroimaging techniques, should be implemented in order to clarify long-term effects of PCU on both neurophysiological and neurocognitive point of view.

## 5. Conclusions

Taken together, our data would seem to suggest that individuals with PCU could be characterized by a trait-specific dysfunctional interaction between the SN and CEN (specifically between the dACC and right PPC) during RS. This result might reflect certain aspects of PCU such as attentional and emotional processes of cannabis-related thoughts, memories, and craving. Therefore, future investigations relating to the triple network model could provide novel insights into human behavior associated with addiction and substance-related disorder.

## Figures and Tables

**Table 1:** eLORETA coordinates of the triple network.

Brain Network	Anatomical Structure	eLORETA MNI Coordinates ^1^ eLORETA Talairach Coordinates ^1^		
		*x*	*y*	*z*
DMN	mPFC	0	55	25
		0	54	20
	PCC	0	−55	20
		0	−52	21
SN	dACC	0	20	35
		0	21	31
	Left AI	−45	15	−5
		−45	14	−5
	Right AI	50	15	−5
		50	14	−5
CEN	Left dlPFC	−45	20	35
		−45	21	31
	Right dlPFC	40	25	50
		40	27	45
	Left PPC	−40	−70	45
		−40	−66	45
	Right PPC	50	−60	40
		50	−56	40

Note: ^1^ coordinates referred to the ROI centroid; coordinates should be considered approximate due to the uncertain boundaries of the anatomical structures and brain activation patterns.

**Table 2:** Demographic and clinical data of participants (N = 36).

	PCU (N = 12)	Non-PCU (N = 24)	test	*p*
**Variables**				
Age–*M (SD)*	23.33 ± 3.47	21.21 ± 2.70	Z-test = 1.06	0.211
Educational level (years)–M ± SD	16.42 ± 1.51	15.54 ± 1.50	Z-test = 0.83	0.504
Men–N (%)	7 (58.3%)	9 (37.5%)	χ^2^_1_ = 1.41	0.236
Tobacco use in the last 6 months–N (%)	8 (66.7%)	7 (29.2%)	χ^2^_1_ = 4.63	0.031
CAST–*M (SD)*	10.25 ± 4.31	0.00 ± 0.00	-	-
CAGE–*M (SD)*	0.67 ± 1.07	0.04 ± 0.20	Z-test = 0.82	0.504
CAGE ≥ 2–N (%)	3 (25%)	0 (0%)	χ^2^_1_ = 6.55	0.011
SCL-K-9–*M (SD)*	1.22 ± 0.97	0.73 ± 0.44	Z-test = 0.94	0.336

Note: PCU = problematic cannabis users; CAST = Cannabis Abuse Screening Test; CAGE = self-report measure of alcohol use problems; SCL-K-9 = Symptom-Checklist-K-9.

## Abbreviations

eLORETA	exact Low Resolution Electromagnetic Tomography software
MNI	Montreal Neurological Institute
DMN	default mode network
mPFC	medial prefrontal cortex
PCC	posterior cingulate cortex
SN	salience network
dACC	dorsal anterior cingulate cortex
AI	anterior insula
CEN	central executive network
dlPFC	dorsolateral prefrontal cortex
PPC	posterior parietal cortex
